# Composites of Nanoporous Gold and Polymer

**DOI:** 10.1002/adma.201203740

**Published:** 2013-01-03

**Authors:** Ke Wang, Jörg Weissmüller

**Affiliations:** Institut für Werkstoffphysik und Werkstofftechnologie, Technische Universität Hamburg-Harburg21073 Hamburg, Germany E-mail: weissmueller@tuhh.de; Institut für Werkstoffforschung, Werkstoffmechanik, Helmholtz-Zentrum Geesthacht21502 Geesthacht, Germany

Experimental investigations of the strength of small objects – such as micropillars or nanowires – often point towards a trend of increasing strength with decreasing dimension,^1^–^3^ approximating the theoretical shear strength when the size drops to the lower nanometer region.^1^–^4^ The observation of theoretical strength in defect-free crystals, such as whiskers, irrespective of their size exemplifies that the trend of smaller is stronger is related to the defect structure.^5^–^7^ The interaction of dislocations with the surface is another important factor, as is evidenced by in situ observation of large recoverable flow-stress changes during interfacial charging or electrosorption.^8^ Irrespective of its microscopic origin, the high strength at small size suggests a search for design strategies that yield high-strength materials exploiting the mechanical properties of metal nanostructures. A key challenge, namely assembling many (10^18^ for 1 cm^3^ of material with a 10 nm structure size) nanoscale objects into a macroscopic body, can be overcome by synthesis via dealloying.^9^–^11^ The process provides millimeter- or centimeter-sized monolithic samples consisting of a homogeneous network structure of nanoscale “ligaments” with uniform size that can be controlled down to well below 10 nm.^12^, ^14^ Investigations by transmission electron microscopy, focused ion beam imaging, and electron backscatter diffraction have established that nanoporous metals prepared in this way are polycrystalline with a grain size of 10–100 μm.^15^, ^16^ Each micrometer-sized grain is nanoporous, so that neighboring ligaments share the same crystal lattice. In other words, the local structure in volumes of, say, 1 μm^3^, is that of a single crystal containing a contiguous nanoscale pore network. The mechanical behavior of these materials obeys scaling equations derived for foams with macroscopic porosity, and the local strength of the ligaments follows the same^3^, ^17^–^19^ or similar^16^, ^20^ trends as individual nanowires. The material, and in particular nanoporous gold (npg), has thus been studied as a model system for size-effects on the plasticity of nanostructures.

Significantly, npg can be deformed to large plastic strain in compression, in contrast to many other nanomaterials that fail after few percent of deformation.^21^ The deformability of npg may be understood from the macroscopic constitutive behavior. Similar to nanopillars or nanowires,^4^ the individual nanoscale ligaments are expected to show little work hardening. Yet, the absence of transverse plastic strain in the macroscopic nanoporous metal implies that compression under uniaxial stress is completely carried by densification of the network of ligaments.^16^ By virtue of the scaling^22^ of the flow stress, *σ*_F_, with solid volume fraction, *ϕ*, as *σ*_F_ ∝ *ϕ*^3/2^, the densification causes work hardening at the macroscopic scale, promoting stable and uniform^16^ plastic flow in compression. However, the same argument also points at the central deficiency of nanoporous metals as structural materials: plastic flow under *tensile* loading reduces the density, resulting in work softening and, hence, in shear instability and brittle failure.^8^, ^23^ Obviously, ductilization of the material requires, first and foremost, a materials design that prevents the density change under load. Here, we explore impregnation of the nanoporous metal with a polymer as a way towards that goal. We show that impregnation indeed leads to ductilization. The interpenetrating nanocomposite material which is thus formed exhibits a number of unusual and technologically attractive properties, specifically ductility in tension, the option of cold-forming, high electric conductivity and a strength significantly exceeding that of each of the constituent phases.

As described in the Experimental Section, npg samples with different structure size were prepared by alloy corrosion and subsequent thermal coarsening. Vacuum impregnation with a mixture of epoxy resin and amine hardener, followed by curing, yielded the composite samples. Chemisorption of the amine hardener on the gold surface^24^ implies good wetting and, hence, a tendency for spontaneous imbibition of the fluid by the nanoporous metal. According to the Washburn law, the time-scale for imbibition scales with the inverse square-root of the viscosity.^25^, ^26^ In the interest of facile impregnation, a low viscosity was achieved by selecting a small resin chain length and by heating the resin/hardener mixture.

Figure S1 in the Supporting Information displays scanning electron micrographs of the cross-sectional surface of npg-epoxy composites with ligament sizes, *L*, of 15, 50 and 150 nm. Energy-dispersive X-ray fluorescence analysis (EDX) in the scanning electron microscope (SEM) finds a uniform carbon signal on cross-sections of all of the samples, suggesting complete impregnation. This notion is further confirmed by the SEM images of polished cross-sections in **Figure** **1**. The polymer phase is not apparent in the SEM images, but its presence is evidenced by the conservation of the metal nanostructure: the polished surfaces of native npg, Figure 1a, appear dense and featureless in the SEM since polishing destroys the fragile ligament structure. In contrast, the ligament structure is perfectly retained at polished surfaces of the composite, Figure 1b. This results from the stabilization of the metal phase against collapse by the interpenetrating polymer phase. The observation of stable nanoporous microstructures after polishing can only be understood in terms of complete impregnation. Extensive investigations of cross-sectional SEM micrographs revealed no voids in the polymer.

**Figure 1:**
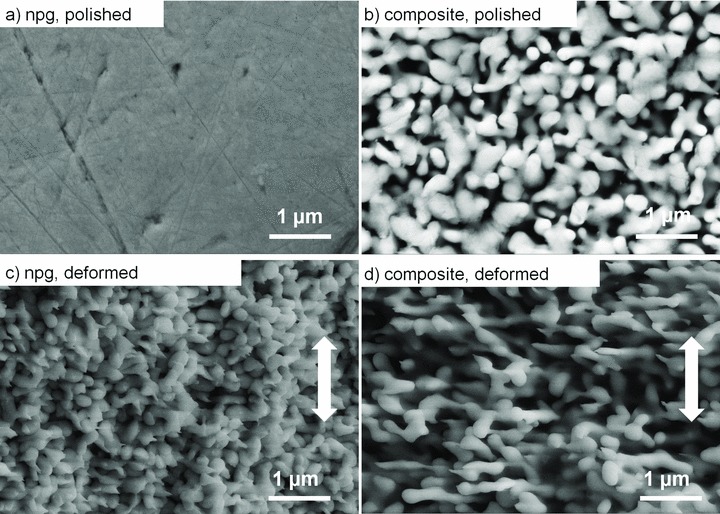
Scanning electron micrographs of cross-sections. Top row: as-prepared materials with a ligament size of 150 nm after polishing: a) nanoporous gold (npg); b) composite. Polishing destroys the microstructure in npg, but not in the composite. Bottom row: materials with a ligament size of 150 nm after compression to engineering strain 0.7 (arrows: compression direction): c) Cross-sectional surface of the compressed npg sample; d) cross-sectional surface of a compressed composite. Note the much stronger densification in npg. Note that ligaments do not break except at the macroscopic cut/fracture surface. The polymer phase is transparent in the micrographs, only the metal is imaged.

**Figure** **2** displays results of microhardness tests on the composite material and on its individual constituent phases. The hardness values for npg agree well with the previously reported trend of increasing *H*_V_ with decreasing *L*. The straight line of best fit in the log-log graph gives a power-law *H*_V_ ∝ *L*^−1.0 ± 0.1^, steeper than some previous data, but in agreement with work by Jin et al.^16^ Note that the samples densify during coarsening, and that increasing strength of metal foams at higher density may partly compensate the decreasing strength of the ligaments at larger *L*.

**Figure 2:**
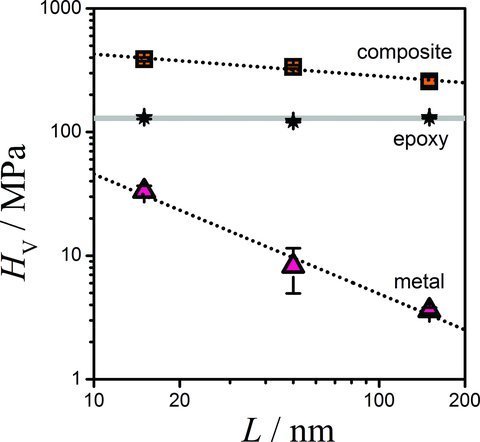
Vickers microhardness, *H*_V_, of nanoporous gold and composite samples versus the ligament size, *L*. Epoxy samples were taken from the same mould as the composites and data is plotted at the respective *L*-value. The dotted lines represent the best linear fits; the grey line is a guide to the eye. The hardness data is tabulated in the Supporting Information.

*H*_V_ data for the epoxy was taken on samples collected from the same mould as the respective composite samples and are displayed at the *L*-value of the respective composite. The data testifies to a well reproducible, constant hardness of the pure epoxy. At *H*_V_ = 129 ± 5, the hardness considerably exceeds that of the nanoporous metal. Nonetheless, the hardness values for the composites are again higher. In fact, at *H*_V_ = 389 ± 4 the hardest composite is threefold harder than the epoxy. Straight-line fits to the composite data give *H*_V_ ∝ *L*^−0.18 ± 0.04^. Even though the size-dependence of the hardness is weaker for the composite than for npg, the data shows that the enhanced strength of the nanoscale ligaments propagates into enhanced composite properties. Remarkably, the hardness of the npg-epoxy composite of any *L* exceeds that of each of its constituent phases. This is contrary to what would be expected based on rule-of-mixture type behavior in a multiphase material.

Figure S2a,b in the Supporting Information show optical micrographs of indents in npg and composite. While cracks surround the indents in npg, those of the composite are free of cracks.

**Figure** **3** shows graphs of true stress versus true (compressive) strain from compression tests on millimeter-sized samples at strain rate 10^−4^ s^−1^ (see also tabulated data in the Supporting Information). Composite samples with *L* = 15 nm typically fail at lesser strain than those with larger ligaments, and also exhibit a somewhat larger sample-to-sample scatter in yield stress. However, it is apparent that all of the materials exhibit excellent deformability in compression. For the npg samples, the yield stress is not resolved, and the plastic flow behavior is characterized by a continuously increasing flow stress, for instance from an initial ≍1 MPa to >150 MPa for *L* = 150 nm samples. The absence of transverse plastic flow, which underlies the work hardening,^20^ is illustrated by the images of npg before and after compression, see inset in Figure 3.

**Figure 3:**
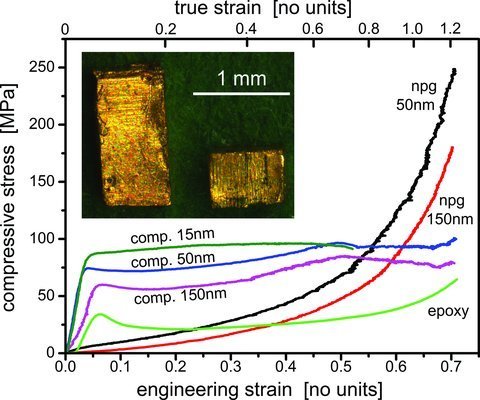
Results of compression tests on cuboid samples of initial dimension ≍1 mm × 1 mm × 1.5 mm. The main graph shows mean true compressive stress versus engineering strain, with the true strain shown on the top abscissa. Tests were performed to an engineering strain of 0.7, corresponding to nearly full density for the nanoporous gold (npg) samples. The inset shows side views of porous metal samples before (left) and after (right) compression to an engineering strain of 0.6, illustrating the absence of transverse plastic flow. Data for yield strength are tabulated in the Supporting Information.

The most obvious features of the compressive stress–strain curve for the composite in Figure 3 are the appearance of a yield point for the *L* = 50 and 150 nm samples and a drastic reduction in work hardening. While the work hardening index varies between *n* = 1 and 0.5 for npg samples in the course of the compression test, the *n*-values for the composite samples for strains sufficiently beyond the yield point are much smaller, never exceeding 6 × 10^−2^ for *L* = 15 nm and 4 × 10^−3^ for the larger *L*.

Consistent with our expectation, the change, Δ*V*, in macroscopic volume is suppressed in the composites. The native npg samples exhibit relative volume shrinkage (density increase) of Δ*V*/*V* ≍ −0.62 ± 0.05 for *L* = 150 nm and Δ*V*/*V* ≍ −0.72 ± 0.04 for *L* = 50 nm during an engineering strain of −0.70, bringing the solid fraction, *ϕ*, after compression close to 1. By contrast, the volume of the composite sample changed only slightly. For a compressive strain of −0.71, we found Δ*V*/*V*_0_ ≍ −0.03 ± 0.01 for diameter of 150 nm and −0.046 ± 0.01 for diameter of 50 nm. A similar volume change, −0.025 ± 0.01 is found for the pure epoxy sample. The slight decrease of the composites' macroscopic volume may be understood as the result of the rearrangement of polymer chains under compressive stress.^27^

Comparison with the data for pure epoxy in Figure 3 suggests that the presence of a yield point in the composite reflects a property of the polymer component. Most importantly, the compression data confirms the trends from the hardness tests, in particular: i) the strengths of both, npg and composite samples, increase with decreasing *L* and ii), the composite material at any value of *L* is much stronger than each of its constituents.

Figure 1c,d show cross-sectional SEM images of npg and composite samples, respectively, after compression. The cross-sectional surface of compressed npg was obtained by bending the sample (after the compression test) with the help of tweezers, inducing brittle fracture. Due to their ductility, the composite samples could not be fractured in this way and had to be cut. It is apparent that the metallic ligaments have not failed during the compression tests. This is consistent with the notion of ductile deformation at the nanoscale. The figure also suggests that impregnation with polymer changes the mode of deformation of the metal. Similar to a conventional metal foam,^22^ the pure nanoporous metal may deform at low stress by local bending of the ligaments. The ensuing densification is apparent when Figure 1c is compared with Figure S1 in the Supporting Information. In contrast to the pure porous metal case, the embedding in a composite matrix forces the ligaments to strain along with an essentially volume-conserving macroscopic flow field. This emphasizes axial elongation or shortening of ligaments as the local deformation modes, as supported by the horizontal texture and the elongated aspect of the ligaments in Figure 1d. As compared to bending, the deformation by elongation or compression may require larger local stresses in the ligaments, enhancing the macroscopic strength of the composite.

As a qualitative test of tensile ductility we performed a 3-point bending experiment with the native npg and the composite material. Photographs of the as-tested samples are shown in **Figure** **4**. In striking contrast to the brittle fracture of native npg (Figure 4a), the composite (Figure 4b) can be deformed with no apparent failure in the compressive or tensile regions of the sample. An evaluation of the bending geometry reveals that the maximum true tensile strain was ≍0.1. The drastic enhancement of tensile ductility confirms our strategy.

**Figure 4:**
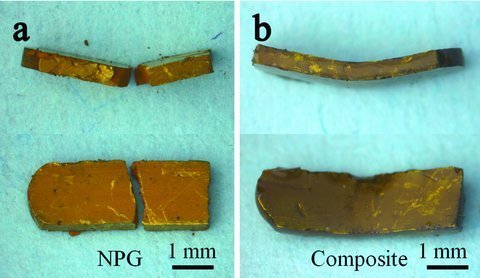
Photographs of native npg (a) and its composite (b) after the three-point bending tests. Samples are shown edge-on and in top view

Resistance measurements on several composite samples with *L* = 150 nm resulted in the electric conductivity value of 260 ± 30 kS m^−1^ at 298 K. At roughly 1% of the conductivity of massive high-purity Cu, this is considerably larger than state-of-the-art composites of epoxy and carbon nanotubes, which reach 13 kS m^−1^.^28^

The present results demonstrate that impregnation with a polymer is an efficient way of reducing the density change during plastic flow of nanoporous metals under a uniaxial load. We had conjectured that suppressing the volume change would also suppress the compression/tension anisotropy of the work hardening. Indeed, the stress–strain curves in compression evidence a drastic reduction of the work hardening for the composite material. Furthermore, while native npg is extremely brittle in tension, the plastic deformation of the composite in three-point bending testifies to its tensile ductility. These findings, along with the high electric conductivity, have several important implications:

1. Our materials design strategy points the way towards implementing the high tensile strength of metal nanowires into a macroscopic structural material. Ligament sizes, *L*, in npg can be tuned down to 4–5 nm,^12^, ^14^ almost a factor 3 smaller than the present material, while the compressive ductility and low mass density are maintained.^16^ Even smaller ligaments sizes have been reached when the surface was covered with an atomic monolayer of stabilizing oxygen species.^13^ Due to its size-dependence, the strength of the metal phase may be enhanced in such porous materials. The strength can be further enhanced by embedding massive dendritic reinforcements in to the porous metal.^29^ Thus, nanoporous-metal-based composite materials may reach higher strength than that which is reported in the present work.
2. It is remarkable that the composite is substantially stronger and harder than each constituent phase individually. As argued above, the finding may partly relate to a change in the mode of deformation of the metal, emphasizing axial flow of the ligaments over bending. Yet, other factors may also contribute, such as strengthening by interfacial phenomena. For the nanoporous metal this has been demonstrated by reversible switching of the strength when npg is wetted by electrolyte and electric charge or adsorbate layers at the surface controlled via the electrode potential.^8^ Bonds with functional groups of the amine hardener may have a similar effect, strengthening the metal on top of the size effect. For the polymer, it is well known that the structure in layers – the “interphase” – near the interface with another solid differs from bulk,^30^, ^31^ and this may further affect the strength. These considerations point toward interesting size effects to be explored in future studies with smaller structure size.
3. Shaping structural – for instance, fiber-reinforced – com-posites for application requires time-consuming and labor-intensive curing in a mould. In contrast, the high ductility of the present material suggests shaping by deep-drawing at ambient temperature, a considerably more convenient procedure. Alloy corrosion may be applied to less costly metals, such as Cu or Ni, opening the way to structural nanocomposites for application that can be manufactured as semifinished product in sheet form and shaped by conventional drawing, forging or machining.
4. Finally, the material achieves a near-metallic conductivity, predestining it for applications where, for instance, static electric charging needs to be avoided, or lightning-strikes tolerated.

Alloy corrosion has already been applied to a wide variety of metals, and can yield nanoporous metals with structure sizes well below what was achieved in our present study. At the same time, polymer science offers a wide parameter space in terms of bonding agents and the possible use of thermoplastic or phase-separating materials. Such studies promise insights into size and interface or interphase effects on the mechanical properties of the metallic and the polymer phases, as well as progress towards a new class of strong, ductile and electrically conductive nanocomposites.

## Experimental Section

Following the synthesis procedures described by Jin et al.,^16^ npg samples were prepared by electrochemical dealloying of arc-melted samples of Ag_75_Au_25_ after homogenization, cutting to size and polishing. After dealloying, the samples were repeatedly rinsed with ultrapure water and dried in vacuum for 3 days. Evaluation of SEM (Leo Gemini 1530) images revealed mean ligament diameters, *L*, of 15 nm in the as-prepared material. Samples with *L* of 50 and 150 nm were prepared by annealing in air at 300 °C for 2 and 30 minutes, respectively.

The solid volume fraction, *ϕ*, was estimated based on the amount of Ag removed and on the macroscopic volume contraction. EDX revealed ≤2 at% Ag in the porous metal, while in situ dilatometry put the relative volume shrinkage during dealloying at ≤2%.^16^ This implied *ϕ* = 0.27 ± 0.01 for the as-prepared porous metal. Dilatometry during the coarsening annealing showed volume shrinkages of <3% and ≍15% for 2 and 30 min annealing, respectively. This implied solid fractions of 0.27–0.30 and 0.42 for the samples with *L* of 50 nm and 150 nm, respectively.

The composites were prepared in a commercial vacuum impregnation unit (CitoVaca, Struers, Germany). In brief, a vacuum vessel containing a mould with the nanoporous metal sample was evacuated, the mould filled in situ with the liquid mixture of epoxy resin and hardener at 55 °C, and the liquid then pushed into the pores by venting the vessel to air. Surplus epoxy was removed by either: a) cutting the sample from the cured epoxy, or b) lifting the sample out of the epoxy before curing. A small chain-length bisphenol-F epoxy resin (BER 20, Buehler, Germany, number-average molecular weight ≤ 700 g mol^−1^) was used and the mixture of epoxy and amine hardener (BEH 20, Buehler, Germany) at mass ratio 4/1 was heated to 55 °C for impregnation.

The Vickers hardness, *H*_V_, was measured using a microhardness tester (VMHT MOT, Uhl, Germany). Loads of 10 g for the composites and 1 g for npg were chosen so as to obtain comparable indentation depths. The indentation time was 30 s. Indent diagonals were visually measured in optical microscopy and converted into hardness. At least 20 indents were made on each of several samples for each measurement. As an estimated error, we have quoted the variance for the set of indents.

Compression tests used a Zwick 1484 testing machine and cuboid samples of initial dimension ≍1 mm × 1 mm × 1.5 mm loaded by planar anvils. The true stress, *σ*, and true strain, *ε*, were computed from the elongation by exploiting the findings of constant volume (for epoxy and composite) or constant cross-section (for npg, see Jin et al.^16^). A feedback loop controlled and progressively reduced the crosshead speed so as to maintain constant true strain rate while the sample length varied. Work-hardening indices, 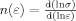, were estimated by taking derivatives on smoothed graphs of ln*σ* versus ln*ε*. Volume changes associated with the compression were estimated by measuring the sample dimensions before and after compression with a toolmaker's microscope (Mitutoyo TM, Germany). A bending test used the testing machine with a custom-build three-point-bending rig.

The electric conductivity of the composite samples was measured by a four-probe approach using a resistance meter (Resistomat, Burster). An appropriate aspect ratio was obtained with samples 4.2 mm × 1.8 mm × 0.6 mm in dimension, contacted at the end surfaces.

## Supplementary material

As a service to our authors and readers, this journal provides supporting information supplied by the authors. Such materials are peer reviewed and may be re-organized for online delivery, but are not copy-edited or typeset. Technical support issues arising from supporting information (other than missing files) should be addressed to the authors.
