# Supplementary material for: Composites of Nanoporous Gold and Polymer
Source: Adv Mater. 2013 Jan 3;25(9):1280–4. doi: 10.1002/adma.201203740 (PMC3613747; doi:10.1002/adma.201203740)
Supplement: Supplementary file 1 [file adma0025-1280-SD1.pdf]

Copyright WILEY-VCH Verlag GmbH & Co. KGaA, 69469 Weinheim, Germany, 2013.

ADVANCED MATERIALS

# Supporting Information

for *Adv. Mater.*, DOI: 10.1002/adma.201203740

## Composites of Nanoporous Gold and Polymer

*Ke Wang and Jörg Weissmüller**

# Supporting Online Material for „Composites of Nanoporous Gold and Polymer”

Ke Wang[1] and Jörg Weissmüller[1,2]

1 - Institut für Werkstoffphysik und Werkstofftechnologie, Technische Universität Hamburg-Harburg, Hamburg, Germany

2 - Institut für Werkstoffforschung, Werkstoffmechanik, Helmholtz-Zentrum Geesthacht, Geesthacht, Germany

**Supplementary figures on microstructure characterization**

This material accompanies the main publication in Reference [1].

Figure S1 gives an illustration of the microstructure of composites based on nanoporous metal with different ligament sizes. The ligament size was varied by coarsening during thermal annealing.

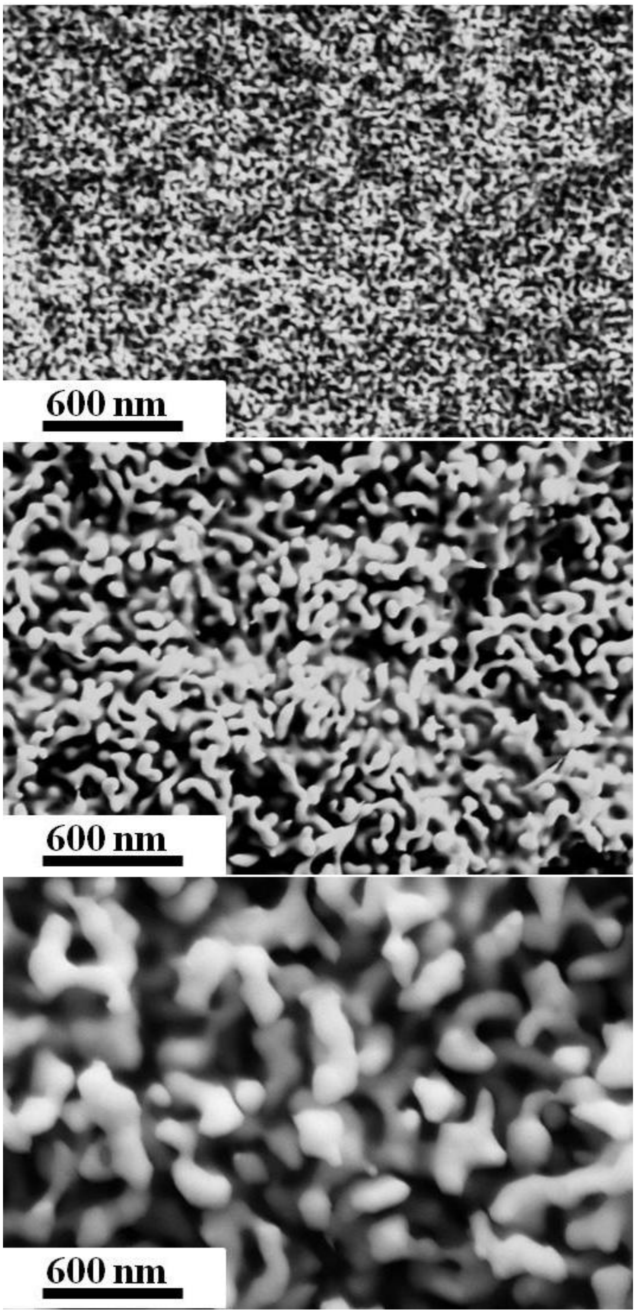


**Figure S1.** Scanning electron micrographs of polished cross-sectional cut surfaces of npg-epoxy composites with ligament diameter of 15, 50 and 150 nm (from top to bottom). Epoxy phase gives no contrast in the SEM and so is not imaged. Note that the ligament structure is conserved in spite of the polishing. This can only be explained by stabilization of the metal microstructure through the interpenetrating epoxy. Integrity of the microstructure on all cross-sections supports complete impregnation.

**Supplementary material on mechanical characterization**

Figure S2 shows exemplary hardness indents from the microhardness tests, results of which are compiled in Fig. 2 of the main text, Ref. [1]. Table 1 compiles numerical values of hardness and yield stress.

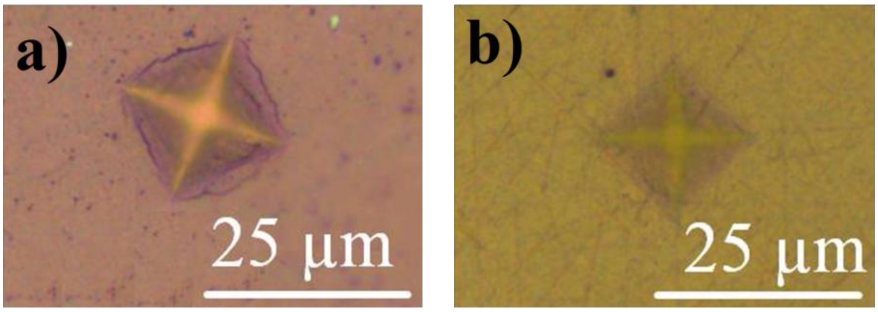


**Figure S2.** Representative optical micrographs showing indentation marks of Vickers microhardness tests. **a)**, as-prepared nanoporous gold (npg); **b)** composite sample. Ligament size is 150 nm. Note cracks in npg and absence of cracks in composite.

**Table S1.** Numerical values of structural and mechanical characteristics of the composite and of its constituents, nanoporous gold (npg) and epoxy resin, as shown in the graphs of Figs. 2 and 3 in the main text, Ref. [1]. $L$ – ligament size; φ - metal volume fraction, $H_V$ - Vickers hardness, $\sigma^Y$ - 0.2% offset yield stress. For the npg samples, yielding is not resolved in the stress-strain curves of Ref. [1], and yield stress values are from data in Fig. 3b) of Ref. [2].

| | $L$ [nm] | φ [no units] | $H_V$ [no units] | $\sigma^Y_{02}$ [MPa] |
|---|---|---|---|---|
| composite | 15 | 0.27±0.01 | 387±14 | 80±10 |
| | 50 | 0.285±0.015 | 336±11 | 66±3 |
| | 150 | 0.42±0.05 | 257±22 | 54±3 |
| npg | 15 | 0.27±0.01 | 33±4 | 26±4 [2] |
| | 50 | 0.285±0.015 | 8.2±3 | 8.6±0.9 [2] |
| | 150 | 0.42±0.05 | 3.6±0.2 | 3.2±0.3 [2] |
| epoxy | — | — | 129±6 | 28±3 |

**References**

[1] K. Wang, J. Weissmüller, *Composites of Nanoporous Gold and Polymer*, Advanced Materials, *under review* (2012).

[2] H. J. Jin, L. Kurmanaeva, J. Schmauch, H. Rosner, Y. Ivanisenko, J. Weissmüller, Acta Materialia 2009, 57, 2665.
